# Genomic Epidemiology and Transmission Dynamics of Global Coxsackievirus B4

**DOI:** 10.3390/v15020569

**Published:** 2023-02-19

**Authors:** Jinbo Xiao, Jianxing Wang, Huanhuan Lu, Yang Song, Dapeng Sun, Zhenzhi Han, Jichen Li, Qian Yang, Dongmei Yan, Shuangli Zhu, Yaowen Pei, Xianjun Wang, Wenbo Xu, Yong Zhang

**Affiliations:** 1 WHO WPRO Regional Polio Reference Laboratory, National Laboratory for Poliomyelitis and National Health Commission Key Laboratory of Biosafety, National Institute for Viral Disease Control and Prevention, Chinese Center for Disease Control and Prevention, Beijing 102206, China; 2 Shandong Center for Disease Control and Prevention, Jinan 250014, China; 3 Laboratory of Virology, Beijing Key Laboratory of Etiology of Viral Diseases in Children, Capital Institute of Pediatrics, Beijing 102206, China; 4 Center for Biosafety Mega-Science, Chinese Academy of Sciences, Wuhan 430071, China

**Keywords:** Coxsackievirus B4, hand, foot, and mouth disease, phylogenetics, recombination, transmission dynamics

## Abstract

The aim of this study was to determine the global genetic diversity and transmission dynamics of coxsackievirus B4 (CVB4) and to propose future directions for disease surveillance. Next-generation sequencing was performed to obtain the complete genome sequence of CVB4, and the genetic diversity and transmission dynamics of CVB4 worldwide were analyzed using bioinformatics methods such as phylogenetic analysis, evolutionary dynamics, and phylogeographic analysis. Forty complete genomes of CVB4 were identified from asymptomatic infected individuals and hand, foot, and mouth disease (HFMD) patients. Frequent recombination between CVB4 and EV-B multiple serotypes in the *3D^pol^* region was found and formed 12 recombinant patterns (A-L). Among these, the CVB4 isolated from asymptomatic infected persons and HFMD patients belonged to lineages H and I, respectively. Transmission dynamics analysis based on the VP1 region revealed that CVB4 epidemics in countries outside China were dominated by the D genotype, whereas the E genotype was dominant in China, and both genotypes evolved at a rate of > 6.50 × 10^−3^ substitutions/site/year. CVB4 spreads through the population unseen, with the risk of disease outbreaks persisting as susceptible individuals accumulate. Our findings add to publicly available CVB4 genomic sequence data and deepen our understanding of CVB4 molecular epidemiology.

## 1. Introduction

Enteroviruses belong to the genus *Enterovirus*, family *Picornaviridae*, and are common pathogens causing childhood diseases [1]. Enteroviruses cause a wide range of clinical symptoms, including hand, foot, and mouth disease (HFMD), febrile rashes, and severe neurological and respiratory complications such as acute flaccid paralysis (AFP), encephalitis, aseptic meningitis, shock, myocarditis [2]. The four enterovirus species associated with the human disease include EV-A, EV-B, EV-C, and EV-D. The six Coxsackievirus group B (CVB) serotypes (CVB1–CVB6) belong to EV-B. CVB3 and CVB5 often cause aseptic meningitis outbreaks [3,4]. Although CVB4 is involved in the pathogenic spectrum of HFMD [5], few related studies exist. Under the effects of genetic recombination and natural selection, enteroviruses, which sometimes receive little attention, cause large-scale disease outbreaks and persistent epidemics. Comprehensive surveillance and robust interventions are essential to control the prevalence and spread of enteroviruses.

The enterovirus genome is a single-stranded, positive-sense RNA with approximately 7400 nucleotides containing a 5′-untranslated region (*UTR*), long open reading frame (*ORF),* and some enteroviruses also contain a short *ORF* and 3′-*UTR* [6]. The genome coding region first forms three polyprotein precursors (*P1*, *P2*, and *P3*); *P1* is further divided into four structural proteins (*VP4*, *VP2*, *VP3*, and *VP1*) while *P2* (*2A*, *2B*, *2C*) and *P3* (*3A*, *3B*, *3C*, *3D^pol^*) form seven non-structural proteins. Serotypes of unknown enteroviruses can be determined using differences in *VP1* sequences [7]. Furthermore, frequent recombination occurs in the *3D^pol^* of different serotypes of the same specie [8], and recombination promotes viral evolution and the ability to adapt to the host and external environments, an important reason why some viruses cause disease outbreaks, despite being rarely noticed [9]. The *3D^pol^*-encoded RNA-dependent RNA polymerases (RdRps) are characterized by low fidelity, so misincorporations are common in viral replication. Recombination may be effective at reducing the accumulation of such errors [10,11], thereby enhancing virus survivability and indirectly leading to the emergence of more pathogenic strains.

In May 2011, an HFMD outbreak was reported in Shandong Province, China, caused by CVB4 [12]. Subsequently, we sequenced and analyzed surveillance specimens from healthy children in Shandong Province in 2010 to understand the hidden circulation and genetic diversity of CVB4 in the population. Using next-generation sequencing (NGS), 40 CVB4 genome sequences were obtained (18 isolated from asymptomatic infected persons and 22 from HFMD patients), contributing nearly half of the GenBank database. Finally, the GenBank database and the newly obtained CVB4 sequences were integrated, and CVB4 epidemiological characteristics, recombination diversity, and Bayesian phylodynamics were determined to identify the possible causes of this outbreak and provide support for related disease surveillance.

## 2. Materials and Methods

### 2.1. Sample Collection, Virus Isolation and Ethical Considerations

To effectively monitor HFMD incidence, a national HFMD pathogen surveillance net was established in mainland China in 2008 for healthy individuals and HFMD patients [13]. The Shandong Center for Disease Control and Prevention collected their clinical samples during 2010–2011 for further identification at the National Polio Laboratory following strict compliance with the Polio Laboratory Manual (4th edition, 2004), published by the World Health Organization. Samples were inoculated in human rhabdomyosarcoma (RD) and human laryngeal epidermoid carcinoma (HEp-2) cell lines, respectively [14]. Cell cultures were collected after complete EV-like cytopathic effects were observed.

All patients or their guardians provided informed consent. The Ethics Review Committee of the National Institute for Viral Disease Control and Prevention (IVDC) of the Chinese Center for Disease Control and Prevention approved the study and confirmed that all methods were performed in accordance with standard guidelines.

### 2.2. RNA Extraction, Library Construction, and NGS

Viral RNA was extracted from cell cultures using a QIAamp Viral RNA Mini Kit (Qiagen, Hilden, Germany), and the RNA concentration was quantified using a Qubit 4 Fluorometer (Thermo Fisher Scientific, Waltham, MA, USA). rRNA was removed using the MGIEasy rRNA Depletion Kit (v1.1, MGI, Shenzhen, China), and the fragmented double-stranded DNA obtained using the MGIEasy RNA Library Prep Set (v3.0, MGI, Shenzhen, China) was subjected to a series of processes such as end repair, dA tailing, adapter ligation, and PCR amplification. Finally, paired-end 100 bp sequencing was performed with the MGISEQ-2000RS High-throughput (rapid) Sequencing Set (v3.1, MGI, Shenzhen, China) and MGISEQ-2000 sequencer (MGI, Shenzhen, China).

Raw data sets were subjected to quality control (QC) using fastp software (v0.21) [15], including removing adapters, discarding short reads < 50 bp in length, cutting low-quality bases (quality scores < 30), and using FastQC (v0.11.8) to check whether they passed QC. Human-associated genomic data with an end-to-end alignment pattern were removed using Bowtie 2 (v2.3.4.3) [16]. The data were selected for assembly using Trinity (v2.9.1) and MEGAHIT (v1.2.9) [17,18], and both assemblies’ results were compared using QUAST (v5.0.2) to select the optimal assembly result [19].

### 2.3. CVB4 Dataset Construction

After excluding clones, the passaged experimental strains, and *ORF* containing many missing or N bases, 40 near-full-length CVB4 sequences (length limited to 6000–7600 nucleotides) were available as of 20 May 2022 in the GenBank database. Combined with the 40 newly sequenced sequences, a genome-wide dataset containing 80 sequences was used for the CVB4 phylogenetic and recombination diversity analyses (Supplementary Table S1). Moreover, *VP1* sequences of CVB4 in the GenBank database were searched (length limited to 700–1000 nucleotides, as of 20 May 2022) and combined with this study’s sequences: a dataset of 225 *VP1* sequences (40 were obtained by sequencing in this study, and the remaining 185 were obtained from the GenBank database) including the time and region which were finally formed. Muscle software (v3.8.31_i86linux32) was used for alignment, while RAxML software (v8.2.12) was used to construct a maximum likelihood (ML) tree of 225 *VP1* sequences [20,21] (Supplementary Figure S1). Finally, the temporal structure of 225 CVB4 VP1 sequences was tested by TempEst (v1.5) and TipDatingBeast packages (R package), and among them, 186 CVB4 *VP1* sequences were selected for a Bayesian phylodynamics study (Supplementary Table S2). Based on the temporal and regional distribution, 60 sequences were selected for phylogeographic analysis (Supplementary Table S3). The sequences were named as follows: Chinese isolates, the isolate number or GenBank number/two-letter provincial abbreviation/CHN/year of collection/genotype or lineage (e.g., 22/SD/CHN/2011/E for the sequence numbered 22 isolated in 2011 in Shandong, China, which belongs to the E genotype in the *VP1* region); isolates from other countries: the GenBank number/three-letter country code/year of collection/genotype or lineage (e.g., MN590273/FRA/2019/D represents the sequence isolated in France in 2019 with GenBank number MN590273, which belongs to the D genotype in the *VP1* region).

### 2.4. Phylogenetic and Recombination Analysis

Muscle software (v3.8.31_i86linux32), ModelGenerator 0.85 [22], and RAxML software (v8.2.12) were used to align the dataset, calculate the best nucleotide substitution model, and construct the dataset maximum likelihood tree, respectively. BioEdit (v7.0.9.0) was used to obtain the sequences identity matrix [23], and the results were presented in TBtool (v1.098726) as a heat map [24].

Sequence genetic diversity analysis was performed using MEGA (v11.0.11) [25] and the neighbor-joining method. The bootstrap method was used to calculate the sequences within- and between-group mean distances, and the Kimura 2-parameter model was chosen. SimPlot software (v3.5.1) was used for similarity analysis of the *ORF* regions [26].

The *P3* regions of CVB4 isolated from asymptomatic infected persons in 2010 and HFMD clinical patients in 2011 were retrieved as query sequences using the Basic Local Alignment Search Tool (BLAST; https://blast.ncbi.nlm.nih.gov/Blast.cgi (accessed on 22 June 2022)) to obtain 100 sequences with the highest similarity in the nucleotide databases for analyzing potential inter- and intra-type recombinations. The recombination detection program (RDP4, v4.46) was used for preliminary recombination analysis [27], and seven methods, RDP, GENECONV, Chimaera, MaxChi, Bootscan, SiScan, and 3Seq, were selected for recombination detection. Recombination events supported by four or more methods were considered credible. Based on the RDP4 analysis results, SimPlot (v3.5.1) (200-nucleotide window moving in 20-nucleotide steps) was used in combination with the maximum likelihood tree to determine the final plausible recombination events.

### 2.5. Amino Acid Site Variation Analysis

The sequence’s nucleotide and amino acid entropy values were analyzed using the Shannon entropy online analysis tool (http://www.hiv.lanl.gov/content/sequence/ENTROPY/entropy_one.html (accessed on 15 July 2022)). When the nucleotide and amino acid entropy values were >0.8 and >0.6, respectively, the site was considered highly variable [28]. The ratio of non-synonymous to synonymous substitutions (dN/dS) is an important indicator for evaluating the effect of selection pressure on coding genes. On the Datamonkey online analysis platform (http://www.datamonkey.org (accessed on 26 July 2022)), two methods, single likelihood ancestor counting (SLAC) and fast, unconstrained Bayesian approximation (FUBAR), were used to infer the individual amino acid sites subjected to positive selection [29,30], and *p*-values were determined using the method described in Table 1. The crystal structures of *3C* (PDB code 5NFS) and *3D^pol^* (PDB code 4ZPD) of CVB3 were used as templates in the SWISS-MODEL online tool (https://swissmodel.expasy.org(accessed on 5 August 2022)) to construct a CVB4 structural model (not shown). Based on these models, multiple sequence alignment results containing secondary structures were generated using the ESPript (v3.0) online tool (https://espript.ibcp.fr/ESPript/ESPript/index.php (accessed on 5 August 2022)) to analyze amino acid differences [31].

### 2.6. Temporal Dynamics Analysis

The potential recombinant detection of the CVB4 *VP1* datasets was performed using the RDP4 (v4.46) and Simplot Software (v3.5.1). TempEst (v1.5) was used to detect the sequences’ temporal structure [32], while the TipDatingBeast package (R package) was used to perform a date-randomization test [33]. Both methods’ detection results are shown in Supplementary Figure S2. Only sequences that passed the above tests were subjected to temporal dynamics analysis. Bayesian phylogenetic analysis was performed using BEAST (v1.8.4) [34]. ModelGenerator 0.85 was used to obtain the optimal nucleotide substitution model for the dataset. Path Sampling/Stepping-stone was used to compare 15 independent combinations of the clock model and the tree prior [35]; the comparison results are presented in Supplementary Tables S4–S5. Trace software (v1.7.1) was used to check whether the parameters converged, and an effective sample size > 200 was a sign of convergence [36]. The Bayesian maximum clade credibility (MCC) tree was finally generated using TreeAnnotator software (v1.8.4), and the first 10% of sampled trees were removed with the burn-in option, and MCC trees were visualized using FigTree software (v1.4) (http://tree.bio.ed.ac.uk/software/FigTree (accessed on 6 September 2022)).

### 2.7. Geographical Clustering Intensity and Phylogeographic Analysis

The Bayesian tip association significance testing (BaTS) program (v2.0) was commonly used to assess the strength of geographic clustering in the data [37]. Usually, each sequence in the dataset is assigned a character state according to its isolation region. The sample trees generated by BEAST software were processed using BaTS software, and three indicators were obtained to evaluate the strength of clustering, including association index (AI), parsimony score (PS), and maximum monophyletic clade (MC). The first two indicators evaluated the overall statistical significance of the geographic clustering of taxa, while the latter was based on the expected and observed mean clade sizes for each group to measure each group’s strength of the association. A *p*-value < 0.05 was considered a significant association.

Reconstructed spatial transmission patterns of CVB4 are important for virus traceability. In Beast, phylogeography analysis was performed, an asymmetric substitution model was selected, and Bayesian stochastic search variable selection (BSSVS) was used to infer social networks [38]. The migration pathway, posterior probability (PP), and Bayes factor (BF) between the different regions were generated using SpreaD3 (v0.9.7.1) [39], and the supported migration pathway met the criteria of BF > 3 and PP > 0.50 [40]. The number of expected regional state transitions was estimated using Markov jump counts. Population analysis with Reticulate Trees (PopART, v1.7) was used to construct a median-joining haplotype network for CVB4 in different regions [41,42].

## 3. Results

### 3.1. Phylogenetic Analysis Based on CVB4 Whole-Genome Sequences

**Figure 1:**
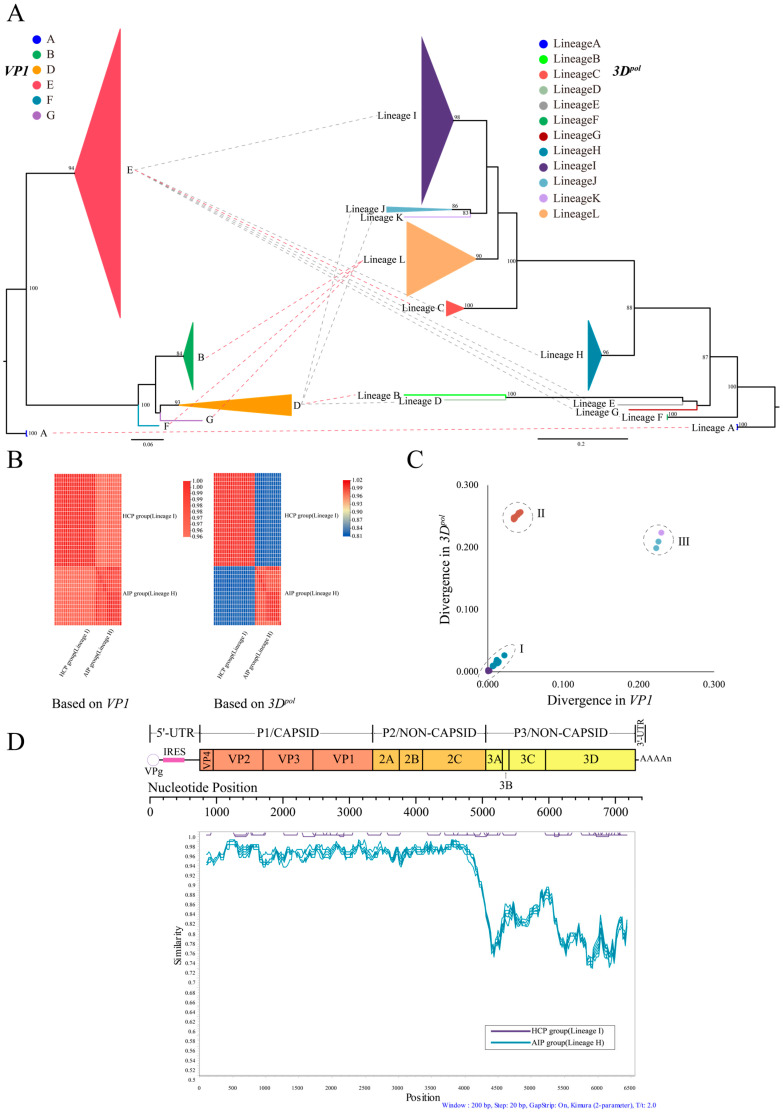
Phylogenetic analysis of CVB4. (**A**) The left and right are the ML tree based on *VP1* regions of 80 CVB4 sequences and *3D^pol^* regions, respectively. Sequences with the same genotype/lineage merged into a triangle, had a size proportional to the number of sequences, and different colors were used to distinguish the different genotypes/lineages. (**B**) Nucleotide similarity heatmap of CVB4 sequences of AIP and HCP groups. The left and right were constructed based on *VP1* and *3D^pol^* sequences, respectively. (**C**) Pairwise distance comparisons of *VP1* and *3D^pol^* sequences, (I) shows the comparison of the CVB4 sequences isolated from AIP and HCP groups in their respective intra-lineage sequences, (II) between CVB4 isolated from AIP and HCP groups, and (III) between lineage -J, -K, and CVB4 isolated from HCP group. (**D**) Similarity comparisons between CVB4 sequences isolated from AIP and HCP groups using sliding window nucleotide similarity analysis, with 200 nucleotide windows moving in steps of 20 nucleotides. We used green lines to represent all sequences isolated from asymptomatic infected persons (AIP group), and purple lines to represent all sequences isolated from clinical patients with HFMD (HCP group).

Overall, forty CVB4 whole-genome sequences were obtained by NGS: 18 from asymptomatic infected persons in 2010 (hereafter referred to as the AIP group) and 22 from HFMD clinical patients in 2011 (hereafter referred to as the HCP group). On average, 3,264,241 reads were obtained from each sample, and the average sequencing depth was 44,141.19 ×. In addition to those newly sequenced in this study, 40 strains were obtained from the GenBank database (including the prototype strain X05690/JVB/NY/USA/1951 isolated in 1951; hereafter, X05690). Therefore, in this study, 80 CVB4 whole-genome sequences were obtained for phylogenetic analysis (Table 2). Compared with the structural protein-coding region, there were large differences in the non-structural protein-coding region of the CVB4 sequence. To visualize these differences, ML trees based on the individual coding regions were constructed (Figure 1A, Supplementary Figure S3). The phylogenetic trees of *VP1* and *3D^pol^* results (Figure 1A) showed that 80 sequences formed only six genotypes in *VP1*, while 12 evolutionary lineages (A, B, C, D, E, F, G, H, I, J, K, and L), were formed in the *3D^pol^* region. Genotype E (lineages C, E, F, G, H, and I) and genotype D (lineages B, D, J, and K) formed six and four evolutionary lineages, respectively. Different genotypes formed the same lineage in the *3D^pol^* region, such as the B and F, while the G genotype formed lineage L. The prototype strain, X05690, was contained in lineage A. Most of the lineages contained infrequent sequences, with the main evolutionary lineages being H, I, and L. Lineages H and I mainly contained sequences isolated from AIP and HCP groups, respectively.

These CVB4 sequences contained in lineages H (isolated from the AIP group) and lineage I (isolated from the HCP group) were classified as genotype E in the *VP1* region but belonged to two completely different evolutionary lineages in *3D^pol^*. This phenomenon was also verified by the heat map of nucleotide similarity between *VP1* and *3D^pol^* (Figure 1B). The nucleotide similarity between the above two datasets in the *VP1* region was very high; however, in the *3D^pol^* region, the sequences of the same lineage had a high similarity with obvious differences between sequences of different lineages. The pairwise distance comparisons results (Figure 1C) showed a positively linearly correlated divergence of intra-lineage sequences in the *VP1* and *3D^pol^* regions of the above two datasets, but when comparing lineages, the divergence between lineage I and lineage-H of the same E genotype increased significantly in the *3D^pol^* region, even surpassing that of lineages K and J, which did not belong to the E genotype. The sliding window nucleotide similarity analysis results of *ORFs* showed a difference between lineages H and I in the coding region (Figure 1D); only the *P1* and *P2* coding regions showed high homology, and the heat map of nucleotide similarity for each partition supported this result (Supplementary Figure S4).

### 3.2. Recombination of CVB4 with Other Enteroviruses

**Figure 2:**
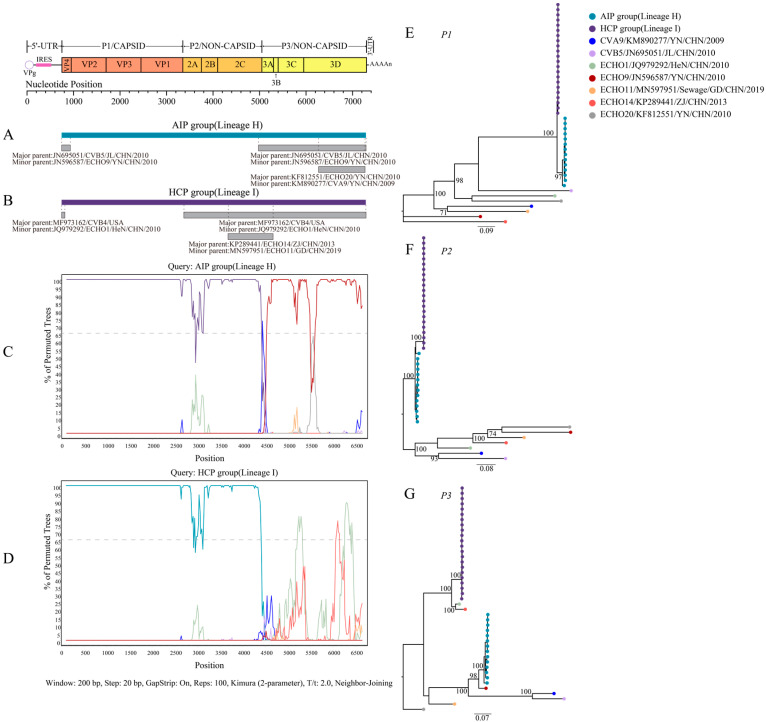
Recombination analysis of CVB4. Recombination analysis results based on sequences obtained after BLAST search are shown in Supplementary Figure S5. (**A**) Recombination analysis results of CVB4 isolated from the AIP group by RDP4. (**B**) Recombination analysis results of CVB4 isolated from HCP group by RDP4. (**C**) Recombination analysis results of CVB4 isolated from the AIP group by Simplot. Bootstrap values above 70% were considered to reflect the presence of recombination events (dashed positions). (**D**) Recombination analysis results of CVB4 isolated from the HCP group by Simplot. (**E**–**G**) ML trees constructed based on *P1*, *P2*, and *P3* regions of the datasets consisting of CVB4 isolated from AIP and HCP groups, and their potential recombination sequences, respectively.

The sequences isolated from the AIP and HCP groups were highly similar in the *P1* and *P2* region sequences but significantly different in the *P3* region, suggesting that the *P3* region may have undergone recombination. Based on the BLAST analysis of the *P3* region, the CVB4 sequences of the AIP and HCP groups had potential recombination with 24 and 26 serotypes, respectively (Supplementary Figure S5). RDP4 further narrowed the scope of recombination events (Supplementary Table S6), and the *VP4* and *2C*-*3D^pol^* regions of CVB5 (JN695051), E9 (JN596587), and *3D^pol^* regions of CVA9 (KM890277), and E20 (KF812551) had recombination events with CVB4 sequences in the AIP group (Figure 2A). The *VP4* and *2A*-*3D^pol^* regions of E1 (JQ979292) and *2C*-3A regions of E11 (MN597951) and E14 (KP289441) had recombination events with the CVB4 sequences of the HCP group (Figure 2B). SimPlot provides more precise results, with bootstrap values > 70% considered a recombination event. The CVB4 sequences of the AIP group had one recombination event in the *P3* region, which was associated with E9 (Figure 2C). Two recombination events occurred in the CVB4 sequences of the HCP group in the *P3* region, with E1 and E14, respectively (Figure 2D). The *ORF* sequences of the potential recombinant strains identified by RDP4 with CVB4 were partitioned into three datasets: *P1*, *P2*, and *P3*. ML trees, based on the above three datasets, were constructed separately (Figure 2E,F). Consistent with the SimPlot results, in the *P1* and *P2* regions, CVB4 sequences of the AIP and HCP groups were clustered together, and in the *P3* region, both formed two different evolutionary branches: CVB4 sequences of the AIP group clustered with E9, and CVB4 of the HCP group clustered with E1 and E14.

### 3.3. Variation Characteristics of CVB4 Amino Acid Position in the P3 Region

**Figure 3:**
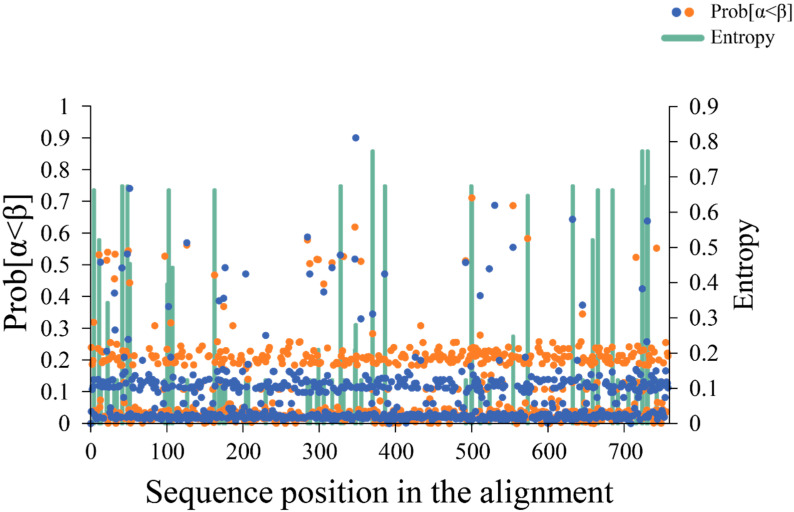
Entropy and pressure selection analysis. The entropy analysis results of the *P3* region of CVB4 isolated from AIP and HCP groups are indicated by green bars, while sites with amino acid entropy values > 0.6 are considered highly variable. The orange and blue circles are the pressure selection results of each amino acid site in the *P3* region of CVB4 isolated from AIP and HCP groups, respectively. Prob [α < β] is the posterior probability of positive selection at a site. Generally, posterior probabilities > 0.9 are strongly suggestive of positive selection.

Shannon entropy analysis showed that the CVB4 sequences of the AIP and HCP groups had 17 amino acid sites with entropy values > 0.6 (Figure 3), indicating that these sites were highly variable. The analysis of nucleotide sequences also identified some highly variable sites (entropy values > 0.8) (Supplementary Figure S6). To analyze the selection pressure on CVB4 of the AIP and HCP groups, two datasets containing the prototype strain X05690 were constructed separately. SLAC results showed that both datasets had a low mean dN/dS ratio (Table 1), indicating that most nucleotides in the CVB4 sequences of the AIP and HCP groups were synonymous substitutions. The FUBAR results showed (Figure 3, Table 1) that the CVB4 of the HCP group had a positive selection in codon 347 (located at *3D^pol^*), and no positive selection sites were found in the CVB4 sequence of the AIP group.

**Figure 4:**
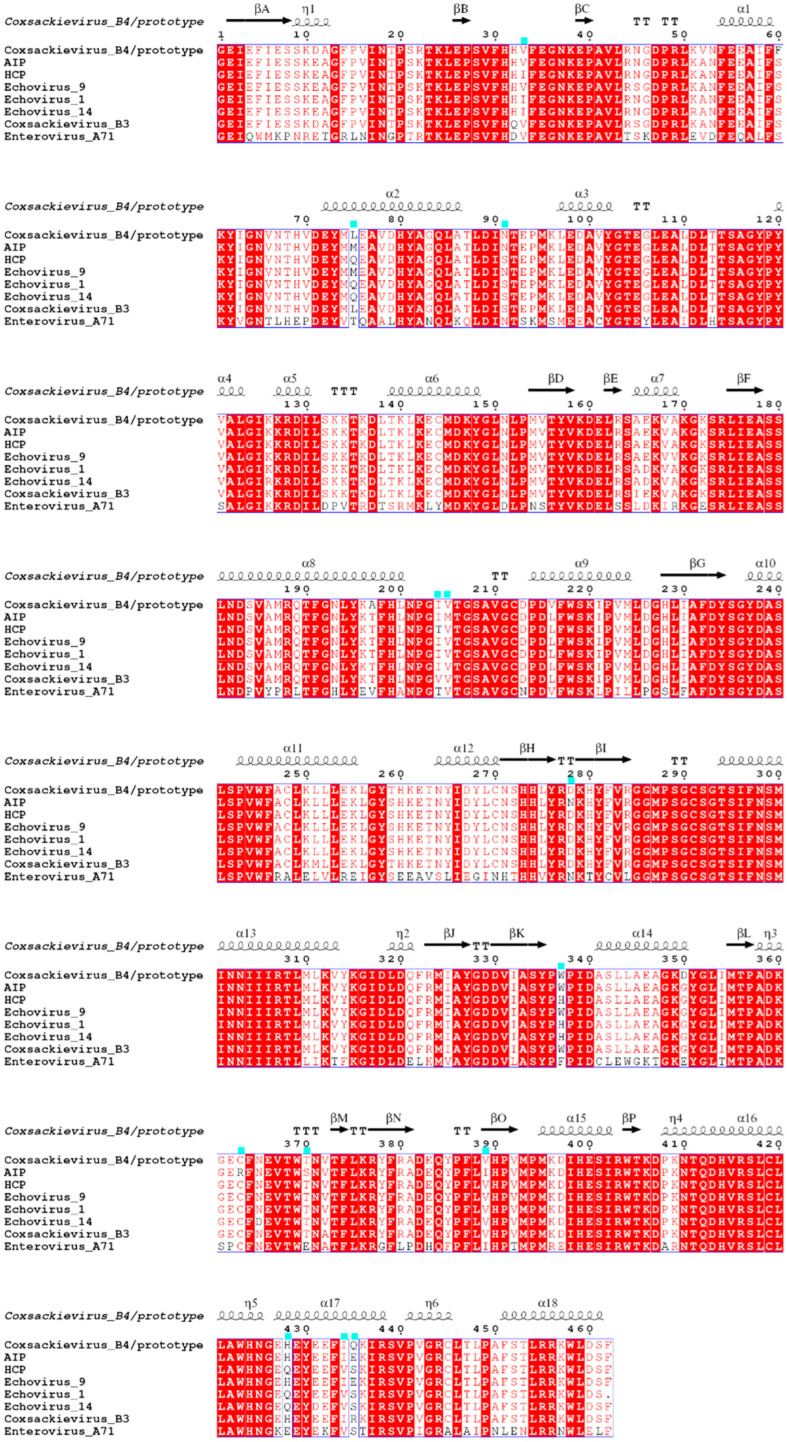
Sequence alignment of CVB4 RNA-dependent RNA polymerase. The enteroviruses involved in the analysis included prototype strains of CVB4, CVB4 isolated from AIP, and HCP groups, E1, E9, E14, CVB3 and EV-A71. The secondary structural elements of CVB4 RdRp (*3D^pol^*) are marked at the top of the alignment; curves and arrows represent α-helices and β-strands, respectively. The number of residues at the top refer to the RdRp (*3D^pol^*) sequence of the CVB4 prototype strain, and cyan square markers represent amino acid sites that differ between AIP and HCP groups.

E9, E1, E14, EV-A71, and CVB3 were added to the structure-based sequence alignment of *3C* and *3D^pol^*. In the *3C* protease-coding region (Supplementary Figure S7), only the amino acids encoded by codon 51 differed between the two datasets, although nucleotide differences were significant. In *3D^pol^* (Figure 4), which encodes an RNA-dependent RNA polymerase, the amino acid differences between the two datasets became apparent, with 13 sites showing differences (codons 33, 75, 91, 204, 205, 278, 337, 363, 370, 389, 428, 434, and 435), with codons 33, 278, and 389 in the BC, HI, and NO loops, respectively. Notably, a phenomenon occurred in most differential sites: the AIP group CVB4 sequences encoded the same amino acids as E9, while that of the HCP group encoded the same amino acids as E1 and E14, which seemed to corroborate the recombination events found previously.

### 3.4. Evolutionary Dynamics of CVB4

**Figure 5:**
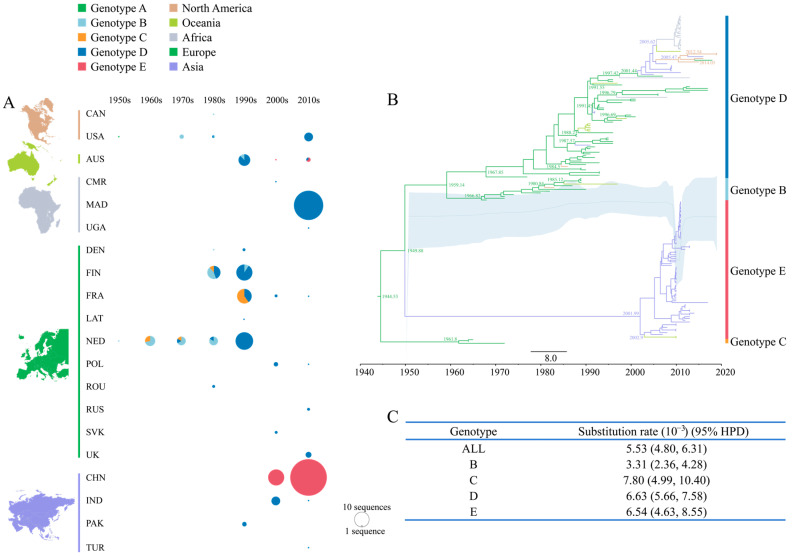
Temporal dynamics analysis of CVB4. (**A**) Geographic and year distribution statistics of 225 CVB4 sequences in this study. The circle size is proportional to the number of sequences (too many CVB4 sequences were isolated in China in the 2010s, the displayed circle size is only one-third of the actual size), and the color of different circles represent different genotypes. (**B**) Maximum clade credibility (MCC) tree based on the *VP1* region of 186 CVB4 sequences. The sequence branches are colored according to different continents. (**C**) Statistics of the substitution rate of the *VP1* region in different CVB4 genotypes.

CVB4-related data from the GenBank database shows that in the past 70 years, CVB4 has been isolated in 20 countries on five continents (Figure 5A), which does not exclude the fact that some countries have underreported or not uploaded surveillance data to the GenBank database due to imperfect surveillance systems. The first two genotypes, B and C, were isolated from the Netherlands in the 1960s and then gradually became prevalent in European countries and remained prevalent for nearly 40 years. Genotype C’s prevalence was limited to Europe, while genotype B was prevalent in North America and Oceania in the late 1980s (Figure 5A,B), with both genotypes disappearing after the 21st century. Genotype D, which also originated in the Netherlands, began to spread to countries in Europe, North America, Africa, Asia, and Oceania in the 1980s and became the most widespread CVB4 genotype. In the 1990s, it replaced the B and C genotypes as a major prevalent CVB4 genotype and remains prevalent today. The E genotype emerged relatively late, was first isolated in China in 2007, was mostly limited to China with only infrequent cases in Australia, and is now a major currently prevalent genotype. The Bayesian skyline plot revealed that the CVB4 population size underwent multiple transformations before 2010 (Figure 5B) and experienced a rapid growth period afterward. By evolutionary rate, the substitution rates of genotypes C, D, and E of >6.50 × 10^−3^ substitutions/site/year, were much higher than that of genotype B at 3.31 × 10^−3^ substitutions/site/year (Figure 5C).

### 3.5. Phylogeographic Analysis of CVB4 Dominant Genotypes

The results of the phylogenetic-trait association analysis (Supplementary Table S7) showed significant statistical tests (*p* < 0.05) for AI, PS, and MC values in most countries, suggesting a significant spatial structure and more localized evolution of D and E genotypes of CVB4 in these regions. The MC statistics of some countries were non-significant (*p* > 0.05) but indicated the existence of geographic mixing for CVB4.

**Figure 6:**
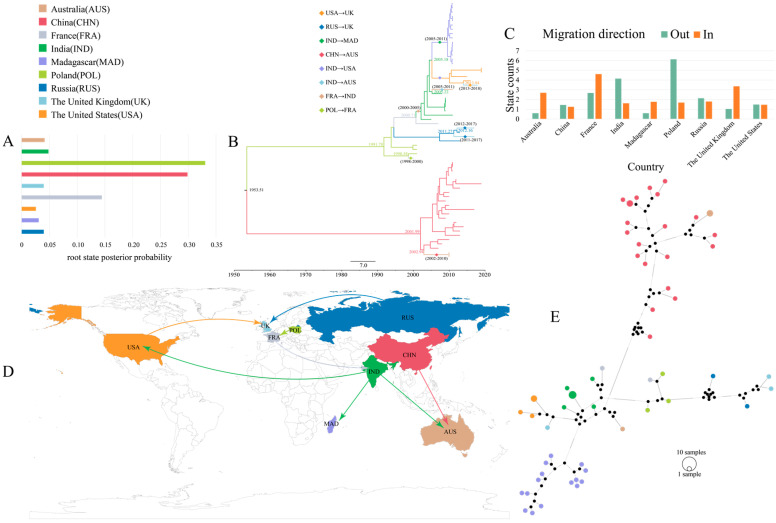
Phylogeographic analysis of CVB4. (**A**) Histogram of the posterior probabilities of the root states of the nine countries. Each country is assigned a different color (used to subsequently distinguish between different countries), with the horizontal and vertical coordinates being the posterior probabilities and different countries, respectively. (**B**) MCC tree of the *VP1* region of 60 CVB4 sequences. The color of the branch represents the inferred location state, and solid diamonds indicate CVB4 spread between different countries, with the inferred years of spread marked nearby. (**C**) Histogram of the total number of inferred location state transitions for the nine countries. (**D**) Spatial diffusion pathways of CVB4 in different countries. Only migration pathways with BF values > 3 and indicators > 0.50 are shown. (**E**) Median-joining haplotype network of 60 CVB4 sequences.

The root state posterior probabilities suggested that the D genotype originated from Poland after 2000 (Figure 6A), whereas China remained the origin of the E genotype. This is consistent with the MCC tree results (Figure 6B), where D and E genotypes evolved independently since 2000, with the D genotype introduced from Poland to France and from France to India in 2000–2005. It began to spread to several countries after 2005, leading to the widespread transmission of CVB4 in Madagascar. Genotype E was only sporadically disseminated from China to Australia during 2002–2010. The inference of the phylogeographic analysis of the migration direction of CVB4 D and E genotypes was consistent with the MCC results (Figure 6D), which were also supported by the BF and posterior probability values (Supplementary Table S8). The state counts results inferred by the Markov jumps method indicated that Poland and India were the major out-migration regions (Figure 6C), while France, Australia, and the United Kingdom were the major in-migration regions.

This relationship can be more clearly determined by constructing a median-joining haplotype network (Figure 6E), and the results showed that the D and E genotypes required undergoing multiple base substitutions to complete mutations, demonstrating that the two genotypes evolved independently. Furthermore, large differences occurred between individuals with the same genotype and between individuals from the same country. Several uncollected but potentially present intermediate individuals were also inferred (Figure 6E, black circles), indicating that a large number of CVB4 were latently present and at some risk of being transmitted.

## 4. Discussion

To the best of our knowledge, this is the first global genome study of CVB4 epidemiology. CVB4, first isolated in 1951, was reported in various countries. During 1959–1998, three genotypes (B, C, and D) were prevalent in Europe and spread to other continents with occasional outbreaks [43]. Currently, the D genotype is gradually replacing other genotypes as the most prevalent genotype, except in China, whereas the E genotype prevalence is almost limited to China.

The causes of CVB4-related disease outbreaks were described using the characteristics of the global CVB4 transmission dynamics. Comparing the CVB4 sequences of the AIP and HCP groups indicated that recombination played a crucial role in CVB4 evolution. From the phylogenetic results for CVB4, 12 lineages were formed in the *3D^pol^* region of the six CVB4 genotypes after different recombination events. The topology of the phylogenetic tree was constructed based on *VP1* and *3Dpol,* which differed significantly, and such recombination events occurred more frequently than in CVA16 [44]. The most frequent recombination occurred in the currently predominant D and E genotypes [12]. Genotype D contained five sequences distributed in Cameroon, Australia, the United States, Romania, and France. The *3D^pol^* region of only five sequences from five countries formed four lineages: lineages B, D, J, and K. Evidently, the D genotype maintains a high frequency of recombination in different regions, which may also be caused by differences in the serotypes of enteroviruses circulating in different regions. Contrarily, the E genotype sequences were mainly distributed in China and very few in Australia. Although the E genotype was endemic in a few countries, as many as five lineages were found in China, which was closely related to the existence of multiple prevalent enterovirus serotypes, indicating that the *3D^pol^* region of CVB4 was characterized by a high recombination frequency. This study’s CVB4 of the AIP and HCP groups was a good example; they were both isolated from Shandong Province and belonged to the same E genotype with highly similar *P1* and *P2* regions but also belonged to lineages H and I in *P3*, respectively.

The recombination analysis results, performed on the CVB4 sequences of the AIP and HCP groups, showed that both underwent completely different recombination events. The former recombined with E9, whereas the latter had a large recombination with E1 and E14 in the *P3* region. This recombination resulted in multiple amino acid site differences between the two groups in *3C* and *3D^pol^* (Figure 4, Supplementary Figure S6), particularly in the *3D^pol^*. The *3D^pol^* region of enteroviruses encodes RNA-dependent RNA polymerase, and amino acid changes may affect the structure, thereby affecting the fidelity of the replicate [10,11], which finally manifests as changes in the virus environmental adaptability and survival. The entropy analysis results also indicated some highly variable amino acid sites in the *P3* region of the CVB4 sequences of the AIP and HCP groups and that CVB4 of the HCP group was positively selected for codon 347 in the *P3* region. After natural selection, sites favorable to the virus were retained and gradually accumulated as dominant sites; the codon 347 site, located exactly in *3D^pol^*, is likely to be a key factor affecting replicase activity.

After nearly 70 years of prevalence, only the D and E genotypes survived. CVB4 prevalence analyses revealed a clear turnover of the dominant genotype. Between 1960 and 1990, genotypes B and C dominated the CVB4 epidemic in Europe. In 1990, the D genotype began to replace them; it also originated in Europe and spread to other continents. Genotypes B and C subsequently disappeared, and genotype D has been prevalent ever since. To explain why the D genotype could be stably prevalent, the *VP1* coding region possesses a high nucleotide substitution rate that greatly increases the possibility of generating adaptive mutation sites, and the E genotype has similar characteristics. After 2000, the D genotype was rarely reported, with some disseminated cases only, during this period. Then, the D genotype was introduced from France to India, where it became endemic and caused acute diarrheal symptoms in children [45]. Then, CVB4 was introduced into Madagascar, the United States, and Australia through intensive population movements, leading to the CVB4 epidemic in Madagascar. The widespread and persistent epidemic suggests that the D genotype is well-adapted to the environment and should be a priority surveillance target. In contrast to the widespread prevalence of the D genotype, the E genotype prevalence was limited to China and caused an associated HFMD outbreak in 2011. The haplotype network analysis results suggest that there are several possible CVB4s that are, as yet, unmonitored, and large differences exist between individuals, which also suggests that the *VP1* region of CVB4 is still mutating at a high rate. Combined with the high-frequency recombination of the *3D^pol^* region, there is sufficient evidence that CVB4 is still evolving toward a better adaptation to its environment and host. It is of great interest whether this will suddenly become one of the major serotypes causing HFMD, such as CVA6, or fatal cases, such as E11.

CVB4 has rarely been reported, and previously, its global epidemiological and transmission characteristics were largely unknown, and its pathogenicity has been significantly underestimated. During 1959–1998, several CVB4 genotypes were widespread in Europe and detected in environmental sewage [43]. Since 2000, with the improvement in enterovirus surveillance systems, CVB4 has been detected in many countries. The national HFMD pathogen surveillance net was established in mainland China in 2008, and CVB4 has been detected since but has not been ascribed to appropriate relevance.

The enhanced surveillance of the prevalence and evolution of CVB4 in China, as well as in other parts of the world, especially for D and E genotypes, is essential for the effective prevention of possible future outbreaks. Although our study was limited by the lack of CVB4-related surveillance, and only relatively few CVB4 whole-genome sequences were obtained, this work provides a foundation for understanding the epidemiological characteristics, phylogenetic features, and Bayesian phylodynamics of CVB4. This study increases the publicly available CVB4 genomic sequence and furthers our understanding of CVB4 molecular epidemiology.

## Figures and Tables

**Table 1:** Pressure selection analysis of the CVB4 *P3* region isolated from healthy children and patients.

Method of Analysis and Dataset	Mean *dN*/*dS*	No. of Negative/Purifying Selection Sites	Position(s) of Positive/Diversifying Selection Site (codon)
SLAC ^a^			
HCP group	0.0161	2	
AIP group	0.0258	6	
FUBAR ^b^			
HCP group		416	347
AIP group		408	

^a^*p*-value threshold of 0.05. ^b^ Posterior probability of 0.9. CVB4, Coxsackievirus B4; *dN*/*dS,* ratio of non-synonymous to synonymous substitutions; SLAC, single likelihood ancestor counting; FUBAR, fast, unconstrained Bayesian approximation; HCP, hand, foot, and mouth diseases clinical patients; AIP, asymptomatic infected persons.

**Table 2:** Information on global CVB4 evolutionary lineages.

Lineage	Genotype	N ^a^	Divergence ^b^	Isolated Countries	Isolated Years
A	A	2	0.002	The United States, Italy	1951
B	D	1	NA ^c^	Romania	1986
C	E	4	0.027	Australia, China	2007–2008
D	D	1	NA	Cameroon	2008
E	E	1	NA	China	2009
F	E	2	0.000	Australia	2010
G	E	1	NA	China	2010
H	E	15	0.019	China	2010, 2013
I	E	34	0.041	China	2010–2014
J	D	2	0.193	Australia, France	2011, 2019
K	D	1	NA	The United States	2016
L	B, F, G	16	0.105	Denmark	NA ^d^

^a^ Number of sequences contained in the evolutionary lineages. ^b^ Overall mean distances, values were calculated from the complete *3D^pol^* region of lineages. ^c^ NA: no value was calculated for lineage with only one sequence. ^d^ NA, GenBank database missing year information. CVB4, Coxsackievirus B4.

## Data Availability

Forty newly sequenced CVB4 whole-genome sequences were uploaded to GenBank (accession numbers OP376489-OP376528) and the China Virus Identification Net (CVIN) (accession numbers CVIN_AA013570-CVIN_AA013609).

## Supplementary Materials

The following supporting information can be downloaded at: https://www.mdpi.com/article/10.3390/v15020569/s1, Figure S1: ML tree of the *VP1* region of 225 CVB4 sequences; Figure S2: Results of root-to-tip regression analysis and date-randomization tests (DRT) on the *VP1* region of the dataset sequence; Figure S3: ML trees of different partitions of 80 sequences; Figure S4: Nucleotide similarity heatmap of CVB4 isolated from AIP group and HCP group; Figure S5: Process of recombination analysis; Figure S6: Results of nucleotide entropy analysis of the CVB4 P3 region isolated from AIP group and HCP group; Figure S7: Sequence alignment of CVB4 encoding the protease (*3C*); Table S1: Information on 80 global CVB4 for phylogenetic analysis; Table S2: Information on 186 CVB4 for Bayesian phylodynamics analysis; Table S3: Information on 60 global CVB4 for phylogeographic analysis; Table S4: Marginal likelihood estimates of the molecular clock model and coalescent model for the 186 CVB4 sequences used for the temporal dynamics analysis; Table S5: Marginal likelihood estimation of the molecular clock model and coalescent model for the 60 CVB4 sequences used for the phylogeographic analysis; Table S6: Recombination results obtained by software RDP4 analysis; Table S7: Analysis of the spatial structure of CVB4 D and E genotypes; Table S8: Statistically supported migration rates of 60 CVB4 based on the *VP1* region.
