# Supplementary material for: Genomic Epidemiology and Transmission Dynamics of Global Coxsackievirus B4
Source: Viruses. 2023 Feb 19;15(2):569. doi: 10.3390/v15020569 (PMC9961479; doi:10.3390/v15020569)
Supplement: Supplementary file 1 [file viruses-15-00569-s001.zip › viruses-2188986-supplementary.pdf]

Genomic Epidemiology and Transmission Dynamics of Global Coxsackievirus B4

Jinbo Xiao [1], Jianxing Wang [2], Huanhuan Lu [1], Yang Song [1], Dapeng Sun [2], Zhenzhi Han [3], Jichen Li [1], Qian Yang [1], Dongmei Yan [1], Shuangli Zhu [1], Yaowen Pei [2], Xianjun Wang [2], Wenbo Xu [1,4] and Yong Zhang [1,4,*]

[1] WHO WPRO Regional Polio Reference Laboratory, National Laboratory for Poliomyelitis and National Health Commission Key Laboratory of Biosafety, National Institute for Viral Disease Control and Prevention, Chinese Center for Disease Control and Prevention, Beijing 102206, China
[2] Shandong Center for Disease Control and Prevention, Jinan 250014, China
[3] Laboratory of Virology, Beijing Key Laboratory of Etiology of Viral Diseases in Children, Capital Institute of Pediatrics, Beijing 102206, China
[4] Center for Biosafety Mega-Science, Chinese Academy of Sciences, Wuhan 430071, China
* Correspondence: yongzhang75@sina.com

Figure S1. ML tree of the *VP1* region of 225 CVB4 sequences. Of the 225 sequences, 40 were obtained by sequencing in this study, and the remaining 185 were obtained from GenBank database. The green part was clade1, which contained the E genotype, and the orange part was clade2, which contained the sequences of B, C, D genotypes. "0.07" is a component of the distance scale of the evolutionary tree, which indicates the unit length of the difference between sequences".

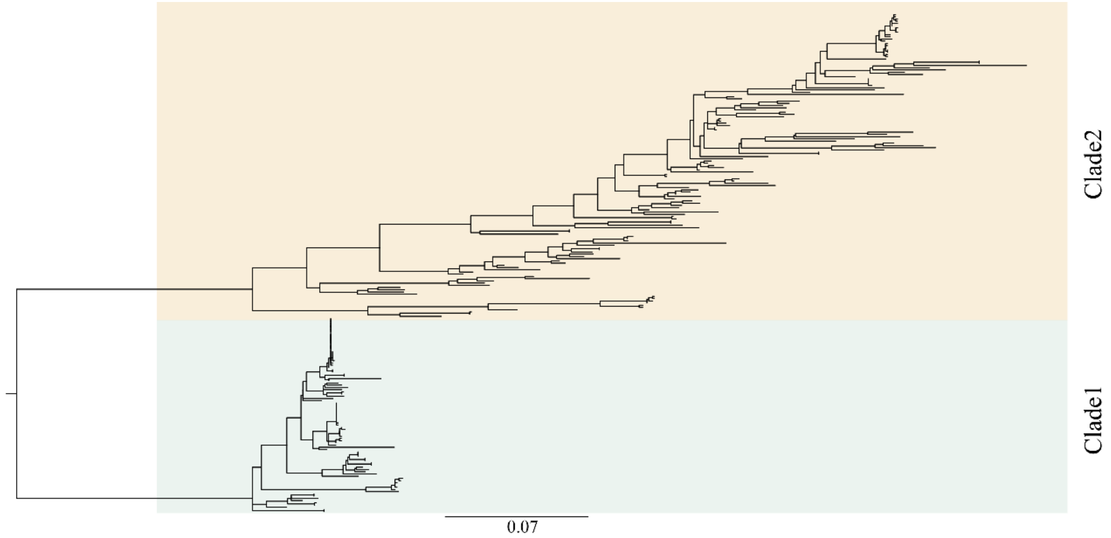

Figure S2. Results of root-to-tip regression analysis and date-randomization tests (DRT) on the *VP1* region of the dataset sequence. The results of the root-to-tip regression analysis were shown on the left, and histograms summarized the frequency of the sequences collected at different times and countries. The DRT results were shown on the right. Only if the 95% credibility interval of the rate estimate does not fall within the 95% credibility intervals of the rate estimates from the date-randomized replicates, the dataset was considered to have sufficient temporal signal for molecular dating. A)186 *VP1* sequences for temporal dynamics analysis. B)60 *VP1* sequences for phylogeographic analysis.

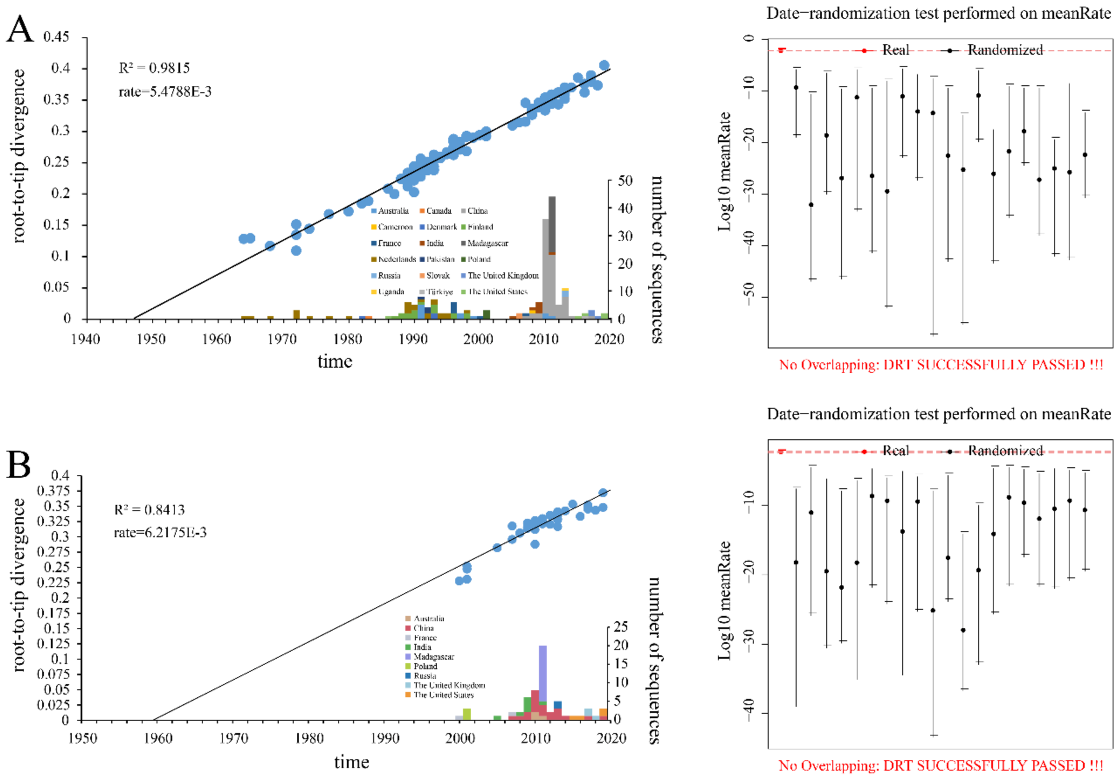

Figure S3. ML trees of different partitions of 80 sequences. Nine diagrams (A-I) contained ML trees of all partitions (*VP2*, *VP3*, *VP4*, *2A*, *2B*, *2C*, *3AB*, *3C* and *3D^pol^*) of 80 sequences except *VP1* (shown in Fig. 1), and each ML tree branch was filled with colors according to different lineages, and branches belonging to the same lineage were indicated by triangles.

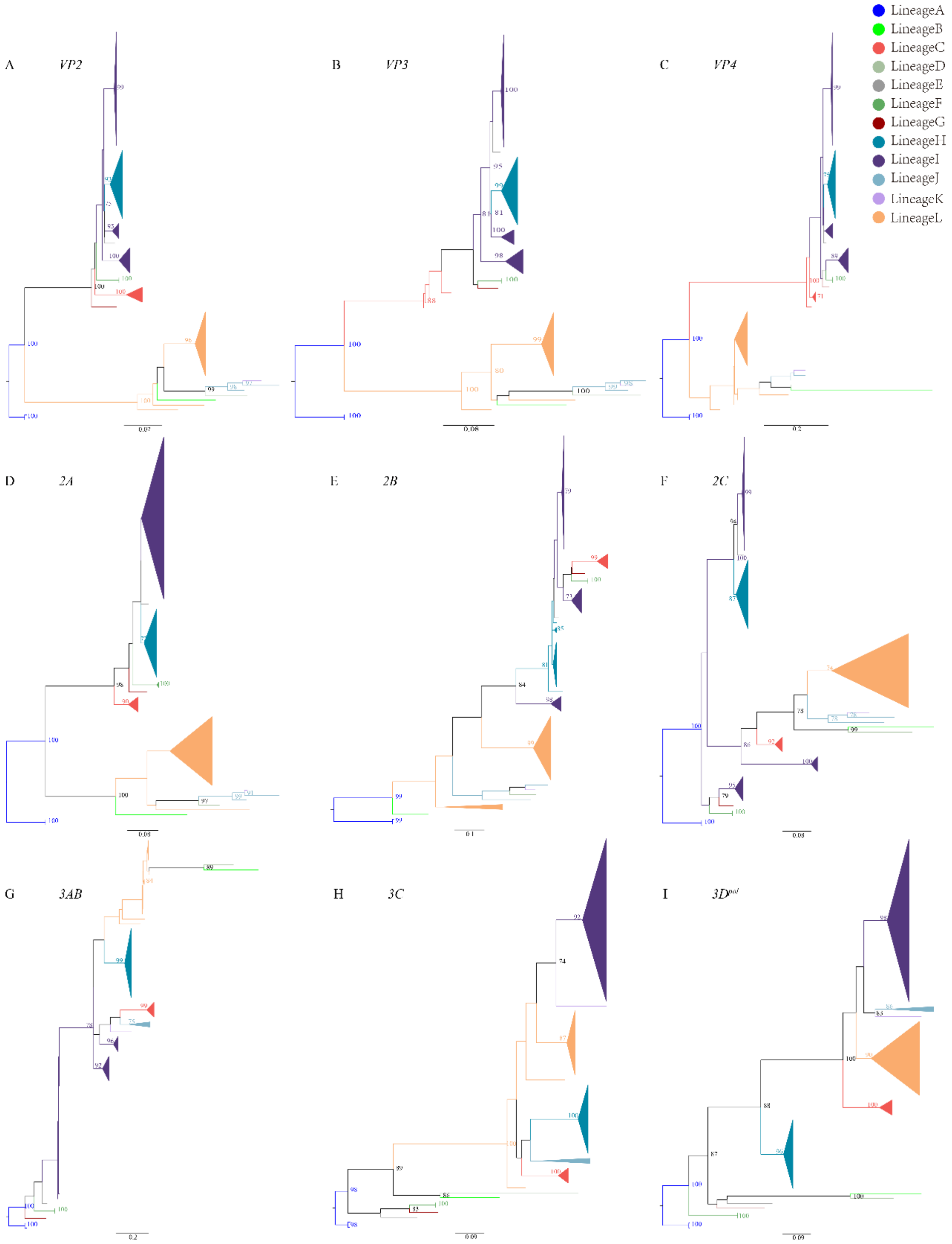

Figure S4. Nucleotide similarity heatmap of CVB4 isolated from AIP group and HCP group. Nucleotide similarity heatmaps of all other partitions except for the *VP1* region (shown in Fig. 1) were displayed in the figure.

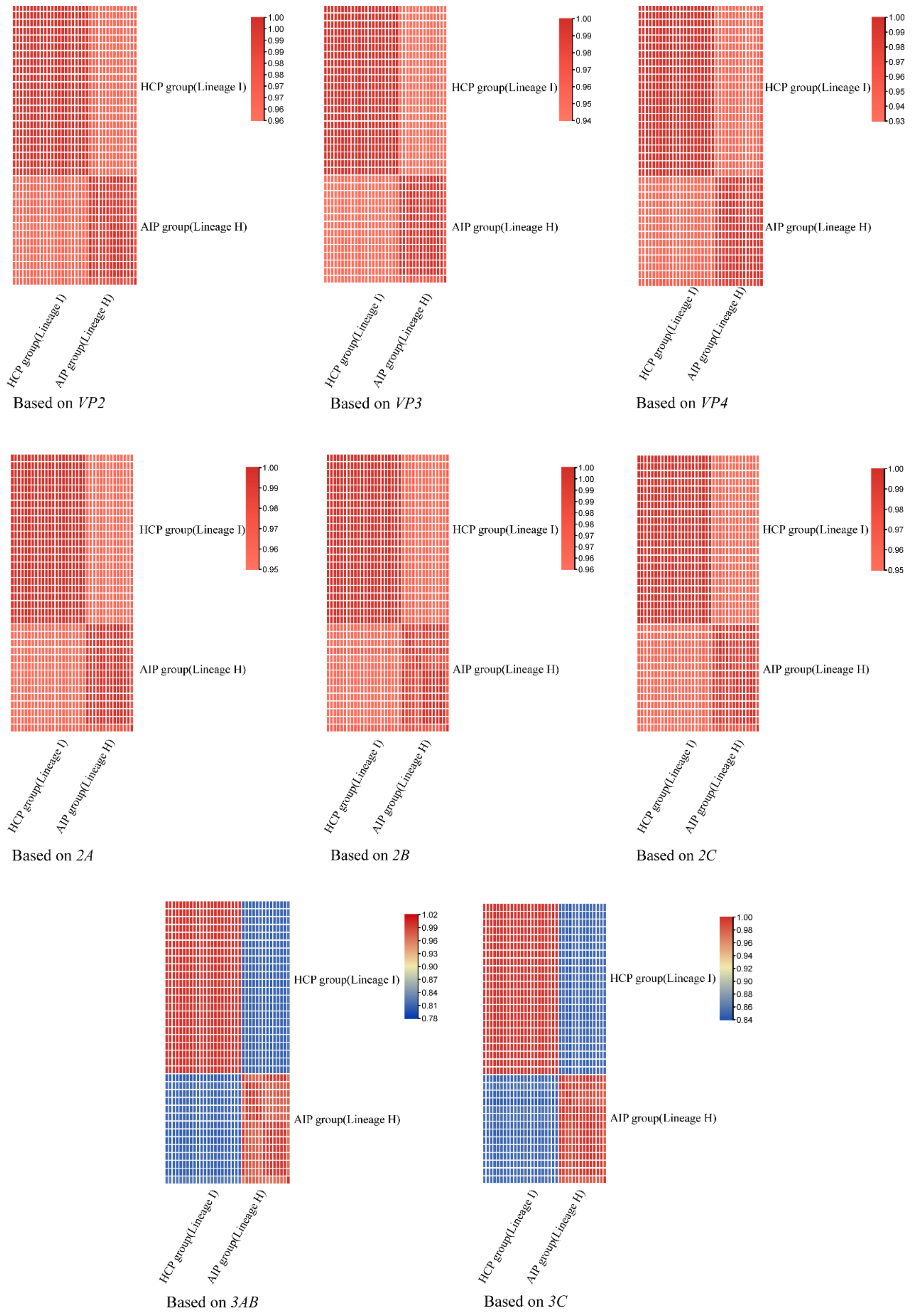

Figure S5. Process of recombination analysis. The recombination analysis process of CVB4 isolated from AIP group and HCP group went through three processes: searching for potential recombinant sequences by BLAST, primary screening of RDP4, and determination by SimPlot.

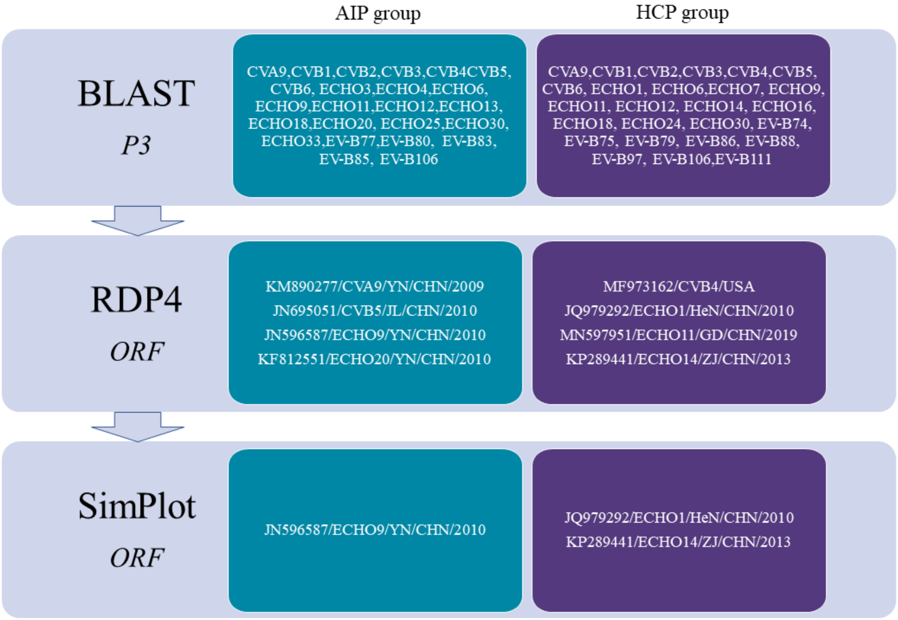

Figure S6. Results of nucleotide entropy analysis of the CVB4 *P3* region isolated from AIP group and HCP group. Nucleotide entropy values greater than 0.8 were considered to be highly variable.

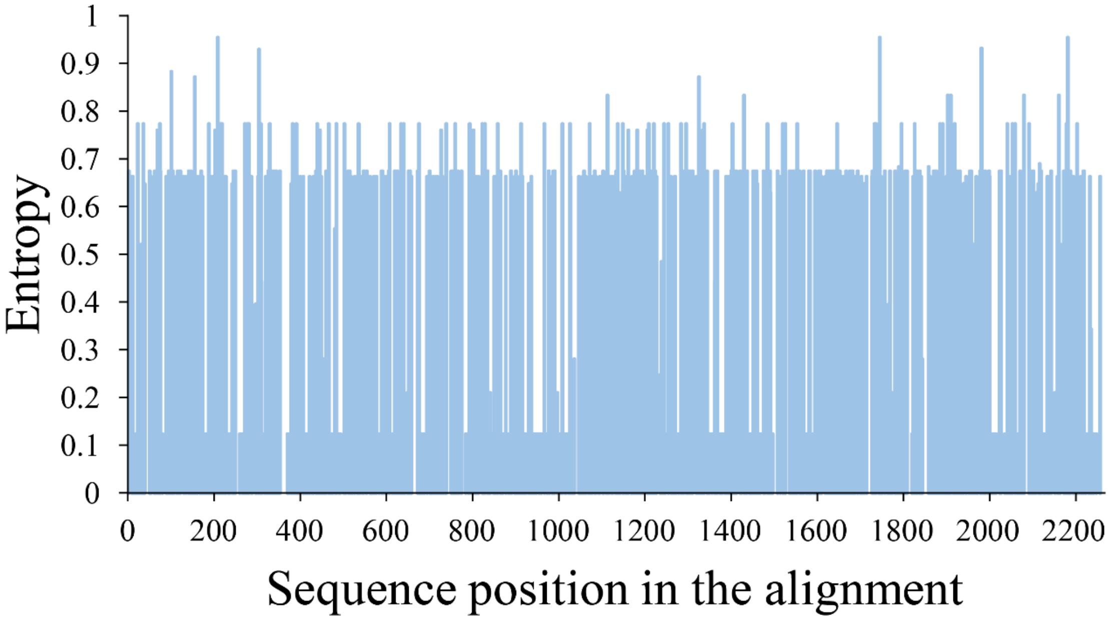

Figure S7. Sequence alignment of CVB4 encoding the protease (*3C*). Enteroviruses involved in the analysis include the CVB4 prototype strain, CVB4 isolated from AIP group and HCP group, E1, E9, E14, CVB3 and EV-A71. The secondary structure elements of CVB4 protease (*3C*) were marked at the top of the alignment; curves and arrows represented α-helices and β-chains, respectively. The number of residues labeled with the sequence of the CVB4 prototype protease (*3C*), and the cyan square marks represented the amino acid sites that differ between AIP group and HCP group.

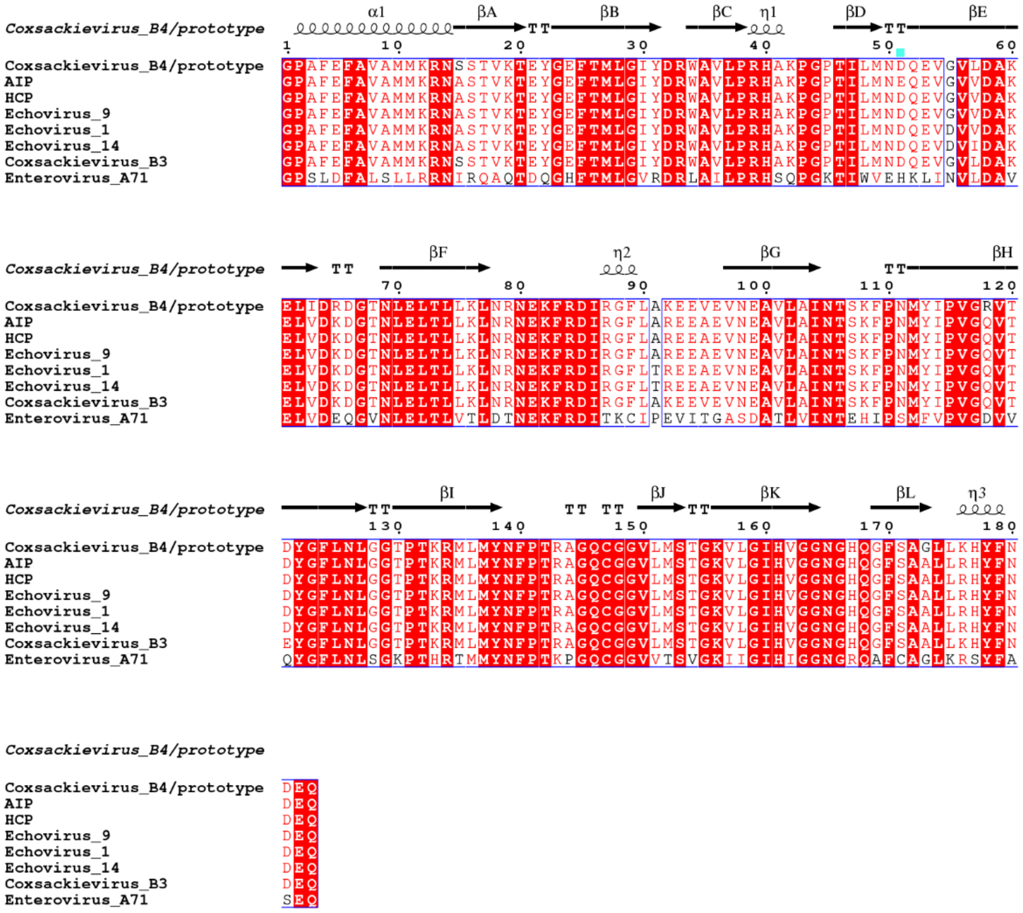

Table S1. Information on 80 global CVB4 for phylogenetic analysis.

Strain name/Genbank No.	Country	Isolated year	Genotype	Evolutionary lineage	Source
DQ480420	Italy	NA	A	A	GenBank
X05690	the United States	1951	A	A	GenBank
LS451289	Romania	1986	D	B	GenBank
MF678347	Australia	2007	E	C	GenBank
MF422559	China	2008	E	C	GenBank
MF422560	China	2008	E	C	GenBank
MF422562	China	2008	E	C	GenBank
JX417724	Cameroon	2008	D	D	GenBank
KM890276	China	2009	E	E	GenBank
MF678300	Australia	2010	E	F	GenBank
MF678319	Australia	2010	E	F	GenBank
KF781524	China	2010	E	G	GenBank
KU566507	China	2013	E	H	GenBank
LYLS201	China	2010	E	H	This study
LYLS213	China	2010	E	H	This study
LYLS216	China	2010	E	H	This study
LYLS258	China	2010	E	H	This study
LYLS328	China	2010	E	H	This study
LYLS332	China	2010	E	H	This study
LYLS345	China	2010	E	H	This study
LYLS359	China	2010	E	H	This study
LYLS361	China	2010	E	H	This study
LYLS365	China	2010	E	H	This study
LYLS369	China	2010	E	H	This study
LYLS372	China	2010	E	H	This study
LYLS413	China	2010	E	H	This study

LYLS436	China	2010	E	H	This study
22	China	2011	E	I	This study
25	China	2011	E	I	This study
27	China	2011	E	I	This study
36R	China	2011	E	I	This study
40	China	2011	E	I	This study
44	China	2011	E	I	This study
46	China	2011	E	I	This study
48	China	2011	E	I	This study
52	China	2011	E	I	This study
54	China	2011	E	I	This study
56	China	2011	E	I	This study
57R	China	2011	E	I	This study
58R	China	2011	E	I	This study
59H	China	2011	E	I	This study
62R	China	2011	E	I	This study
63R	China	2011	E	I	This study
66	China	2011	E	I	This study
67	China	2011	E	I	This study
68	China	2011	E	I	This study
70	China	2011	E	I	This study
71	China	2011	E	I	This study

74	China	2011	E	I	This study
JX018	China	2010	E	I	This study
JX045	China	2010	E	I	This study
JX308222	China	2010	E	I	GenBank
KF781525	China	2010	E	I	GenBank
KP289433	China	2013	E	I	GenBank
KX752784	China	2013	E	I	GenBank
LYLS141	China	2010	E	I	This study
LYLS390	China	2010	E	I	This study
MF179585	China	2013	E	I	GenBank
MF179586	China	2014	E	I	GenBank
MF179587	China	2013	E	I	GenBank
MF179588	China	2013	E	I	GenBank
KF878966	Australia	2011	D	J	GenBank
MN590273	France	2019	D	J	GenBank
KY369904	the United States	2016	D	K	GenBank
KC558559	Denmark	NA	G	L	GenBank
KC558560	Denmark	NA	B	L	GenBank
KC558561	Denmark	NA	B	L	GenBank
KC558562	Denmark	NA	B	L	GenBank
KC558563	Denmark	NA	B	L	GenBank
KC558564	Denmark	NA	B	L	GenBank
KC558565	Denmark	NA	B	L	GenBank
KC558566	Denmark	NA	B	L	GenBank
KC558567	Denmark	NA	B	L	GenBank
KC558568	Denmark	NA	B	L	GenBank
KC558569	Denmark	NA	B	L	GenBank
KC558570	Denmark	NA	B	L	GenBank
KC558571	Denmark	NA	B	L	GenBank
KC558572	Denmark	NA	B	L	GenBank
KC558573	Denmark	NA	B	L	GenBank
AF311939	NA	NA	F	L	GenBank

Table S2. Information on 186 CVB4 for Bayesian phylodynamics analysis.

Strain name/GenBank No.	Country	Isolated year	Genotype	Source
22	China	2011	E	This study
25	China	2011	E	This study
27	China	2011	E	This study
36R	China	2011	E	This study
40	China	2011	E	This study
44	China	2011	E	This study
46	China	2011	E	This study
48	China	2011	E	This study
52	China	2011	E	This study
54	China	2011	E	This study
56	China	2011	E	This study
57R	China	2011	E	This study
58R	China	2011	E	This study
59H	China	2011	E	This study
62R	China	2011	E	This study
63R	China	2011	E	This study
66	China	2011	E	This study
67	China	2011	E	This study
68	China	2011	E	This study
70	China	2011	E	This study
71	China	2011	E	This study
74	China	2011	E	This study
AF159998	Netherlands	1965	C	GenBank
AF159999	Netherlands	1964	C	GenBank
AF160000	Netherlands	1972	C	GenBank
AF160008	Netherlands	1977	D	GenBank
AF160010	Denmark	1993	D	GenBank
AF160011	Denmark	1993	D	GenBank
AF160012	Finland	1993	D	GenBank
AF160014	Finland	1990	D	GenBank
AF160015	Finland	1989	D	GenBank
AF160016	France	1996	D	GenBank
AF160017	France	1996	D	GenBank
AF160018	Pakistan	1992	D	GenBank
AF160019	Pakistan	1992	D	GenBank
AF160020	Netherlands	1993	D	GenBank
AF160021	Netherlands	1993	D	GenBank
AF160022	Netherlands	1994	D	GenBank
AF160023	Netherlands	1994	D	GenBank
AF160024	France	1996	D	GenBank
AF160025	Finland	1998	D	GenBank

AF160026	Finland	1998	D	GenBank
AF160027	Netherlands	1998	D	GenBank
AF160028	Finland	1996	D	GenBank
AF160029	Netherlands	1992	D	GenBank
AF160030	Netherlands	1990	D	GenBank
AF160031	Finland	1989	D	GenBank
AF160032	Netherlands	1989	D	GenBank
AF160033	The United States	1986	D	GenBank
AF160034	Finland	1993	D	GenBank
AF160035	Finland	1990	D	GenBank
AF160036	Netherlands	1995	D	GenBank
AF160037	Netherlands	1995	D	GenBank
AF160038	Finland	1996	D	GenBank
AF160039	France	1996	D	GenBank
AF160040	Finland	1988	D	GenBank
AF160041	Finland	1988	D	GenBank
AF160042	Netherlands	1992	D	GenBank
AF160043	Netherlands	1990	D	GenBank
AF160044	Netherlands	1991	D	GenBank
AF160045	Finland	1991	D	GenBank
AF160046	Finland	1993	D	GenBank
AF160047	Pakistan	1991	D	GenBank
AF160049	Netherlands	1980	B	GenBank
AF160050	Netherlands	1974	B	GenBank
AF160051	Netherlands	1972	B	GenBank
AF160052	Netherlands	1968	B	GenBank
AF160053	Netherlands	1972	B	GenBank
AF160055	Netherlands	1989	B	GenBank
AF160056	Netherlands	1989	B	GenBank
AF160057	Netherlands	1989	B	GenBank
AF160060	Finland	1987	B	GenBank
AF160061	Canada	1983	B	GenBank
AF160062	Denmark	1982	B	GenBank
AF160064	Finland	1990	B	GenBank
FJ868288	Australia	1991	D	GenBank
FJ868289	Australia	1991	D	GenBank
FJ868330	Australia	1991	D	GenBank
FJ868331	Australia	1991	D	GenBank
FJ868332	Australia	1991	D	GenBank
GU142875	Australia	1997	B	GenBank
GU142876	Australia	1997	D	GenBank
GU142877	Australia	1999	D	GenBank
HF948089	France	2000	D	GenBank
HF948090	France	2007	D	GenBank

JF794741	China	2010	E	GenBank
JN016524	China	2010	E	GenBank
JX018	China	2010	E	This study
JX045	China	2010	E	This study
JX308222	China	2010	E	GenBank
JX417724	Cameroon	2008	D	GenBank
JX513577	India	2009	D	GenBank
JX513579	India	2009	D	GenBank
JX513580	India	2009	D	GenBank
JX513581	India	2009	D	GenBank
JX513583	India	2008	D	GenBank
KC867091	China	2008	E	GenBank
KC867092	China	2008	E	GenBank
KC867093	China	2009	E	GenBank
KF177118	India	2011	D	GenBank
KF412921	India	2005	D	GenBank
KF781519	China	2007	E	GenBank
KF781520	China	2010	E	GenBank
KF781521	China	2010	E	GenBank
KF781522	China	2010	E	GenBank
KF781523	China	2010	E	GenBank
KF781524	China	2010	E	GenBank
KF781525	China	2010	E	GenBank
KF878966	Australia	2011	D	GenBank
KJ933328	China	2013	E	GenBank
KJ933329	China	2013	E	GenBank
KM890276	China	2009	E	GenBank
KP289433	China	2013	E	GenBank
KR232695	Madagascar	2011	D	GenBank
KR232711	Madagascar	2011	D	GenBank
KR232712	Madagascar	2011	D	GenBank
KR232730	Madagascar	2011	D	GenBank
KR232731	Madagascar	2011	D	GenBank
KR232732	Madagascar	2011	D	GenBank
KR232739	Madagascar	2011	D	GenBank
KR232743	Madagascar	2011	D	GenBank
KR232751	Madagascar	2011	D	GenBank
KR232758	Madagascar	2011	D	GenBank
KR232760	Madagascar	2011	D	GenBank
KR232761	Madagascar	2011	D	GenBank
KR232763	Madagascar	2011	D	GenBank
KR232775	Madagascar	2011	D	GenBank
KR232776	Madagascar	2011	D	GenBank
KR232778	Madagascar	2011	D	GenBank

KR232782	Madagascar	2011	D	GenBank
KR232792	Madagascar	2011	D	GenBank
KR232793	Madagascar	2011	D	GenBank
KR232796	Madagascar	2011	D	GenBank
KU189236	Poland	2001	D	GenBank
KU189240	Poland	2001	D	GenBank
KU189242	Poland	2001	D	GenBank
KU566507	China	2013	E	GenBank
KU841463	Russia	2013	D	GenBank
KU841464	Russia	2013	D	GenBank
KX752784	China	2013	E	GenBank
KY369904	The United States	2016	D	GenBank
LC412980	China	2017	E	GenBank
LYLS141	China	2010	E	This study
LYLS201	China	2010	E	This study
LYLS213	China	2010	E	This study
LYLS216	China	2010	E	This study
LYLS258	China	2010	E	This study
LYLS328	China	2010	E	This study
LYLS332	China	2010	E	This study
LYLS345	China	2010	E	This study
LYLS359	China	2010	E	This study
LYLS361	China	2010	E	This study
LYLS365	China	2010	E	This study
LYLS369	China	2010	E	This study
LYLS372	China	2010	E	This study
LYLS390	China	2010	E	This study
LYLS413	China	2010	E	This study
LYLS436	China	2010	E	This study
MF179585	China	2013	E	GenBank
MF179586	China	2014	E	GenBank
MF179587	China	2013	E	GenBank
MF179588	China	2013	E	GenBank
MF678300	Australia	2010	E	GenBank
MF678319	Australia	2010	E	GenBank
MG451808	The United Kingdom	2017	D	GenBank
MG845888	The United States	2015	D	GenBank
MG922519	China	2012	E	GenBank
MG922520	China	2012	E	GenBank
MG922521	China	2012	E	GenBank
MG922522	China	2012	E	GenBank
MG922523	China	2012	E	GenBank
MH685712	Uganda	2013	D	GenBank
MK044541	Türkiye	2016	D	GenBank

MN896919	The United States	2019	D	GenBank
MN896920	The United States	2019	D	GenBank
MT109013	China	2010	E	GenBank
MT109015	China	2010	E	GenBank
MT109017	China	2010	E	GenBank
MT109022	China	2010	E	GenBank
MT109023	China	2010	E	GenBank
MT109024	China	2010	E	GenBank
MT109026	China	2010	E	GenBank
MT641357	The United Kingdom	2017	D	GenBank
MT641411	The United Kingdom	2018	D	GenBank
MW390788	Slovak	2006	D	GenBank
MW390789	Slovak	2006	D	GenBank

Table S3. Information on 60 global CVB4 for phylogeographic analysis.

Strain name/GenBank No.	Country	Isolated year	Genotype	Source
22	China	2011	E	This study
25	China	2011	E	This study
71	China	2011	E	This study
HF948089	France	2000	D	GenBank
HF948090	France	2007	D	GenBank
JF794741	China	2010	E	GenBank
JX018	China	2010	E	This study
JX513577	India	2009	D	GenBank
JX513579	India	2009	D	GenBank
JX513580	India	2009	D	GenBank
JX513581	India	2009	D	GenBank
JX513583	India	2008	D	GenBank
KC867092	China	2008	E	GenBank
KC867093	China	2009	E	GenBank
KF177118	India	2011	D	GenBank
KF412921	India	2005	D	GenBank
KF781519	China	2007	E	GenBank
KF781520	China	2010	E	GenBank
KF878966	Australia	2011	D	GenBank
KJ933328	China	2013	E	GenBank
KM890276	China	2009	E	GenBank
KP289433	China	2013	E	GenBank
KR232695	Madagascar	2011	D	GenBank
KR232712	Madagascar	2011	D	GenBank
KR232730	Madagascar	2011	D	GenBank
KR232731	Madagascar	2011	D	GenBank

KR232739	Madagascar	2011	D	GenBank
KR232743	Madagascar	2011	D	GenBank
KR232758	Madagascar	2011	D	GenBank
KR232761	Madagascar	2011	D	GenBank
KR232763	Madagascar	2011	D	GenBank
KR232775	Madagascar	2011	D	GenBank
KR232776	Madagascar	2011	D	GenBank
KR232778	Madagascar	2011	D	GenBank
KR232782	Madagascar	2011	D	GenBank
KR232793	Madagascar	2011	D	GenBank
KR232796	Madagascar	2011	D	GenBank
KU189236	Poland	2001	D	GenBank
KU189240	Poland	2001	D	GenBank
KU189242	Poland	2001	D	GenBank
KU566507	China	2013	E	GenBank
KU841463	Russia	2013	D	GenBank
KU841464	Russia	2013	D	GenBank
KY369904	The United States	2016	D	GenBank
LC412980	China	2017	E	GenBank
LYLS141	China	2010	E	This study
LYLS258	China	2010	E	This study
LYLS436	China	2010	E	This study
MF179586	China	2014	E	GenBank
MF678300	Australia	2010	E	GenBank
MF678319	Australia	2010	E	GenBank
MG451808	The United Kingdom	2017	D	GenBank
MG845888	The United States	2015	D	GenBank
MG922519	China	2012	E	GenBank
MG922522	China	2012	E	GenBank
MN896919	The United States	2019	D	GenBank
MN896920	The United States	2019	D	GenBank
MT641357	The United Kingdom	2017	D	GenBank
MT641411	The United Kingdom	2018	D	GenBank
MW179456	China	2019	E	GenBank

Table S4. Marginal likelihood estimates of the molecular clock model and coalescent model for the 186 CVB4 sequences used for the temporal dynamics analysis. The best-fitting model combination was highlighted in bolded font.

Molecular clock model	Coalescent tree prior	PS	SS
Strict clock	Bayesian SkyGrid	-14070.2	-14068.2
Strict clock	Bayesian Skyline	-14047.7	-14053.4
Strict clock	Constant Size	-14074.2	-14083.2
Strict clock	Exponential Growth	-14070.5	-14068.0
Strict clock	GMRF Bayesian Skyride	-14135.7	-14157.6
Uncorrelated relaxed clock(exponential)	Bayesian SkyGrid	-14116.2	-14126.4
Uncorrelated relaxed clock(exponential)	Bayesian Skyline	-14110.7	-13782.1
Uncorrelated relaxed clock(exponential)	Constant Size	-14125.8	-14136.3
Uncorrelated relaxed clock(exponential)	Exponential Growth	-14116.9	-14127.1
Uncorrelated relaxed clock(exponential)	GMRF Bayesian Skyride	-14172.9	-14198.6
Uncorrelated relaxed clock(lognormal)	Bayesian SkyGrid	-14076.8	-14089.6
Uncorrelated relaxed clock(lognormal)	Bayesian Skyline	-14049.2	-14054.5
**Uncorrelated relaxed clock(lognormal)**	**Constant Size**	**-14070.7**	**-13589.3**
Uncorrelated relaxed clock(lognormal)	Exponential Growth	-14070.7	-14067.0
Uncorrelated relaxed clock(lognormal)	GMRF Bayesian Skyride	-14133.0	-14135.9

Table S5. Marginal likelihood estimation of the molecular clock model and coalescent model for the 60 CVB4 sequences used for the phylogeographic analysis. The best-fitting model combination was highlighted in bolded font.

Molecular clock model	Coalescent tree prior	PS	SS
Strict clock	Bayesian SkyGrid	-6587.3	-6581.5
Strict clock	Bayesian Skyline	-6589.4	-6583.0
**Strict clock**	**Constant Size**	**-6589.5**	**-6578.5**
Strict clock	Exponential Growth	-6590.3	-6587.2
Strict clock	GMRF Bayesian Skyride	-6614.3	-6608.5
Uncorrelated relaxed clock(exponential)	Bayesian SkyGrid	-6617.6	-6607.8
Uncorrelated relaxed clock(exponential)	Bayesian Skyline	-6616.6	-6611.1
Uncorrelated relaxed clock(exponential)	Constant Size	-6617.9	-6611.0
Uncorrelated relaxed clock(exponential)	Exponential Growth	-6618.6	-6613.6
Uncorrelated relaxed clock(exponential)	GMRF Bayesian Skyride	-6624.9	-6620.0
Uncorrelated relaxed clock(lognormal)	Bayesian SkyGrid	-6593.2	-6587.0
Uncorrelated relaxed clock(lognormal)	Bayesian Skyline	-6589.4	-6580.1
Uncorrelated relaxed clock(lognormal)	Constant Size	-6589.9	-6585.7
Uncorrelated relaxed clock(lognormal)	Exponential Growth	-6590.0	-6587.2
Uncorrelated relaxed clock(lognormal)	GMRF Bayesian Skyride	-6610.6	-6606.1

Table S6. Recombination results obtained by software RDP4 analysis.

Strains	Breakpoint		Major parent	Minor parent	Region of recombination	*P*-value of the methods						
	Beginning breakpoint	Ending breakpoint				RDP	Geneconv	BootScan	MaxChi	Chimaera	SiScan	3Seq
AIP group	0	52-192	JN695051/ CVB5/JL/ CHN/2010	JN596587/ ECHO9/YN/CHN/2010	*VP4*	3.420×10^{-30}	1.901×10^{-27}	NA	3.472×10^{-19}	1.764×10^{-15}	2.985×10^{-31}	NA
	4229-4349	6217-6549	JN695051/ CVB5/JL/ CHN/2010	JN596587/ ECHO9/YN/CHN/2010	*2C,3AB,3C, 3D^pol^*	3.420×10^{-30}	1.901×10^{-27}	NA	3.472×10^{-19}	1.764×10^{-15}	2.985×10^{-31}	NA
	5534-5565	6504-6535	KF812551/ ECHO20/YN/ CHN/2010	KM890277/ CVA9/YN/ CHN/2009	*3D^pol^*	3.791×10^{-4}	2.506×10^{-3}	NA	6.917×10^{-6}	4.713×10^{-6}	2.780×10^{-5}	7.985×10^{-5}
HCP group	0	22-94	MF973162/ CVB4/USA	JQ979292/ ECHO1/HeN/CHN/2010	*VP4*	2.185×10^{-36}	4.486×10^{-27}	NA	1.186×10^{-25}	9.434×10^{-28}	3.057×10^{-28}	4.218×10^{-49}
	2577-2593	6549	MF973162/ CVB4/USA	JQ979292/ ECHO1/HeN/CHN/2010	*2A,2B,2C,3AB,3C,3D^pol^*	2.185×10^{-36}	4.486×10^{-27}	NA	1.186×10^{-25}	9.434×10^{-28}	3.057×10^{-28}	4.218×10^{-49}
	3568-3601	4529-4530	KP289441/ ECHO14/ZJ/CHN/2013	MN597951/ ECHO11/GD/CHN/2019	*2C,3AB*	1.837×10^{-11}	NA	NA	3.021×10^{-9}	1.825×10^{-13}	NA	1.402×10^{-2}

Table S7. Analysis of the spatial structure of CVB4 D and E genotypes.

Statistic	observed mean (95% HPD CIs)[d]	null mean (95% HPD CIs)	*P*-value
AI[a]	1.06(0.80,1.27)	5.70(5.01,6.26)	<0.001
PS[b]	11.91(11.00,12.00)	34.98(32.40,37.19)	<0.001
MC[c] (China)	19.30(18.00,21.00)	2.33(1.71,3.95)	0.01
MC (France)	1.00(1.00,1.00)	1.00(1.00,1.00)	1
MC (India)	4.00(4.00,4.00)	1.26(1.00,2.00)	0.02
MC (Australia)	2.00(2.00,2.00)	1.06(1.00,1.60)	0.05
MC (Madagascar)	15.00(15.00,15.00)	1.89(1.02,3.00)	0.01
MC (Poland)	2.00(2.00,2.00)	1.04(1.00,1.17)	0.02
MC (Russia)	1.00(1.00,1.00)	1.00(1.00,1.00)	1
MC (The United States)	2.41(2.00,3.00)	1.03(1.00,1.17)	0.02
MC (The United Kingdom)	1.09(1.00,2.00)	1.03(1.00,1.09)	1

[a]AI, association index.

[b]PS, parsimony score.

[c]MC, maximum monophyletic clade.

[d]HPD CIs, highest posterior density confidence intervals.

Table S8. Statistically supported migration rates of 60 CVB4 based on the *VP1* region.

From	To	Mean migration rate	BF[a]	Indicator
The United States	The United Kingdom	1.17	93.87	0.93
Russia	The United Kingdom	1.61	40.53	0.85
India	Madagascar	1.12	36.62	0.83
China	Australia	0.83	34.13	0.82
India	The United States	1.14	30.87	0.81
India	Australia	1.13	25.99	0.78
France	India	1.14	21.06	0.74
Poland	France	1.34	20.54	0.74

[a]BF, Bayes factor. The BF value > 3 and indicator > 0.50 of statistically supported migration rates are shown.
